# Longitudinal behavioral changes and factors related to reinforced risk aversion behavior among patients with chronic kidney disease during the COVID-19 pandemic

**DOI:** 10.1038/s41598-022-19787-0

**Published:** 2022-09-22

**Authors:** Min Woo Kang, Yaerim Kim, Inae Lee, Hyunwoong Park, Jae Yoon Park, Jung Nam An, Kyung Don Yoo, Yong Chul Kim, Na-Youn Park, Younglim Kho, Kyungho Choi, Jung Pyo Lee, Jeonghwan Lee

**Affiliations:** 1 Department of Internal Medicine, Seoul National University Hospital, Seoul, Republic of Korea; 2 Department of Internal Medicine, Keimyung University School of Medicine, Daegu, Republic of Korea; 3 School of Public Health, Seoul National University, Seoul, Republic of Korea; 4 Department of Laboratory Medicine, Seoul National University Boramae Medical Center, Seoul, Republic of Korea; 5 Department of Internal Medicine, Dongguk University Ilsan Hospital, Goyang, Republic of Korea; 6 Department of Internal Medicine, Hallym University Sacred Heart Hospital, Anyang, Republic of Korea; 7 Department of Internal Medicine, Ulsan University Hospital, University of Ulsan College of Medicine, Ulsan, Republic of Korea; 8 Department of Internal Medicine, Seoul National University College of Medicine, Seoul, Republic of Korea; 9 Department of Health, Environment and Safety, Eulji University, Seongnam, Republic of Korea; 10 Department of Internal Medicine, Seoul National University Boramae Medical Center, 20, Boramae-Ro 5-Gil, Dongjak-Gu, 07061 Seoul, South Korea

**Keywords:** Psychology and behaviour, Kidney diseases, Risk factors

## Abstract

In patients with chronic kidney disease (CKD), coronavirus disease 2019 (COVID-19) has a higher mortality rate than the general population; therefore, prevention is vital. To prevent COVID-19 infection, it is important to study individuals’ risk aversion behavior. The objective of this study was to understand how the behavioral characteristics of physical distancing, hygiene practice, and exercise changed in patients with CKD during the COVID-19 pandemic and to identify the characteristics of patients who showed weakened or strengthened behavioral changes. We analyzed data from the Study on Kidney Disease and Environmental Chemicals (Clinical Trial No. NCT04679168), that examined a prospective cohort of patients with CKD. This cohort included patients with CKD who visited the participating hospitals for the first time between June and October 2020 and the second time between October 2020 and January 2021. Data on demographics, socio-economic details, and behavioral characteristics were collected through a questionnaire survey. Using a multivariable logistic regression model, we identified whether COVID-19 infection risk perception and previous strong behavioral changes affected behavioral changes during the first and second visits. A total of 277 patients (33.2% females) were included in the analysis. Nine out of 12 behaviors were reinforced at the first visit, and five out of nine reinforced behaviors were weakened at the second visit. A high-risk perception of COVID-19 infection was not associated with the tendency of overall behavioral reinforcement or maintaining behaviors in an enhanced state at the second visit. Strong behavioral changes at the patients’ first visit to the hospital were associated with a tendency to strengthen or maintain reinforced behaviors at the second visit (adjusted odds ratio 1.99, 95% confidence interval 1.19–3.34; *P* = 0.009). Even if the initial COVID-19 risk perception is high, behavioral changes worsen over time. Individuals who showed more active behavioral changes at the beginning of the COVID-19 pandemic tended to maintain reinforced behavior over time. Continuous education and monitoring are needed to maintain changed behaviors, especially in patients with a high initial COVID-19 risk perception.

## Introduction

The coronavirus disease-2019 (COVID-19) pandemic is a rapidly spreading global health threat. Owing to its high infectivity and fatality and the absence of direct antiviral drugs, various social and public health interventions for COVID-19 are necessary^1^. Many non-pharmaceutical interventions have been effective in preventing the spread of COVID-19^2–5^. However, implementing physical distancing and strengthening hygiene practices have led to drastically changed lifestyles and involve some inconvenience and sacrifice^6,7^.

For the lasting success of public health policy, appropriate risk perception and sustained voluntary behavioral changes are required^8–10^. One example is the national “lockdowns.” These legally enforceable measures can be effective in preventing the spread of COVID-19 in the short term, but they have a negative impact on the quality of life and national economies, making them difficult to maintain over the long term^11,12^. While COVID-19 risk perception has a direct effect on behavioral changes, there is limited information on their duration. In addition, there is a lack of research on factors that can sustain behavioral changes.

The mortality rate of COVID-19 has been reported to be 0.2–15% across countries^13^. This rate is especially high in older adults or those with comorbidities such as chronic kidney disease (CKD)^14–17^. Patients with CKD who contracted COVID-19 had a higher risk of hospitalization, severe infection, intensive care unit admission, and mortality than those without CKD^18^. In addition, COVID-19 affects the psychological well-being of patients with CKD^19^. Therefore, patients with CKD must stringently adhere to preventive measures including physical distancing and hygiene practices. These measures are particularly important in countries with high population density, such as South Korea^20^.

Most studies have only investigated the immediate effects of physical distancing and behavioral changes in the general population; therefore, it is valuable to show how risk aversion behaviors change over time in high-risk groups such as patients with CKD^21,22^. Thus the purpose of this study was to determine how behavioral characteristics such as physical distancing, hygiene practices, and exercise have changed in patients with CKD since the spread of COVID-19, and to identify the characteristics of people who showed weakened or strengthened behavioral changes. These behavioral changes during the follow-up period were compared with baseline levels before the COVID-19 pandemic. The change in exercise frequency was included in the analysis as a modifiable behavioral factor during the COVID-19 pandemic. As people generally exercise in public places such as fitness clubs, reduced exercise levels were interpreted as a physical distancing-related behavior. Factors associated with the maintenance of reinforced behavioral changes after COVID-19 were also analyzed.

## Materials and methods

### Study participants and design

The data were obtained from the Study on Kidney Disease and Environmental Chemicals (SKETCH; Clinical Trial No. NCT04679168), a prospective cohort study of patients with CKD. The inclusion criteria for the SKETCH cohort were as follows: (1) adults aged 19 years or older, (2) CKD as the main diagnosis, and (3) having been treated twice or more in an outpatient department of kidney medicine for more than 3 months. The cohort enrolled CKD patients with an estimated glomerular filtration rate (eGFR) of ≥ 15 and < 60 mL/min/1.73 m^2^ or (2) eGFR ≥ 60 mL/min/1.73 m^2^ and urine protein/creatinine ratio (uPCR) of > 0.3 g/g. The exclusion criteria were as follows: (1) being followed up for fewer than 3 months before enrollment, (2) having a recent history of acute kidney injury, progressive malignancy, cerebral infarction, cerebral hemorrhage, or myocardial infarction, and (3) undergoing hemodialysis or taking immunosuppressants.

This study began in June 2020 and patients were recruited from five tertiary university hospitals: Seoul National University Boramae Medical Center, Keimyung University Dongsan Hospital, Dongguk University Ilsan Hospital, Hallym University Sacred Heart Hospital, and Ulsan University Hospital. Eligible patients with CKD were screened before their regular visit to the nephrology clinic, and written informed consent was obtained from the attending physician. A total of 309 adult patients were enrolled and scheduled to be followed up at 3-month intervals till the end of the COVID-19 pandemic. This study included patients with CKD who visited the participating hospitals for the first time between June and October 2020 and the second time between October 2020 and January 2021.

### Questionnaire and clinical data

In the SKETCH, trained surveyors conducted a questionnaire survey for participating patients on their first and second visits to the hospital. Demographic data, such as age and sex, along with socio-economic and behavioral characteristics (e.g., income status, smoking status, and alcohol consumption) were collected at the first visit. In addition, questions on the perceived risk of COVID-19 infection and patients’ health levels were included. The risk perception of COVID-19 infection was determined on a five-point Likert scale ranging from “never” (1) to “extremely high” (5); patients selecting 4 or 5 were classified as having a high-risk perception. Similarly, health level perception was determined using a five-point Likert scale ranging from “very bad” (1) to “very good” (5), and patients selecting 3 or higher were classified as having good health perception. For both the first and second visits, the survey inquired about implementing physical distancing, hygienic behavior, and physical activity before the COVID-19 pandemic and at the time of the first and second visits. Questions related to physical distancing included visits to public places (number of visits/week), use of public transportation (number of times/week), use of private vehicles (hours/day), and duration of home stay (hours/day). Questions on hygienic behaviors inquired about the frequency of handwashing over 30 s (times/day) and showering (times/day), use of face masks (times/week) and hand sanitizer (number of uses/week), frequency of doing laundry (times/week), clothes used before laundry (numbers), and home cleaning (times/week). Patients were also asked about the frequency of exercise over 30 min and maintaining regular exercise to determine the level of physical activity. Exercise frequency was determined on a four-point Likert scale: (1) 1–2 times per week, (2) 3–4 times per week, (3) 5–6 times per week, and (4) almost daily. In the above 12 behavioral characteristics grouped under physical distancing, hygiene practice, and exercise, excluding maintaining regular exercise, patients with five or more behavioral changes that reinforced physical distancing and hygiene practice or those who engaged in less exercise compared to the pre-COVID-19 data were defined as the strong behavioral change group. The mean number of confirmed COVID-19 cases in Korea from a week to a day before the patients’ second visit was also considered using data from the Korea Centers for Disease Control and Prevention.

Anthropometric data (height and weight), blood pressure, and laboratory data were collected at both visits. Kidney function was assessed based on eGFR calculated using the Chronic Kidney Disease Epidemiology Collaboration equation. Advanced CKD was defined as an eGFR of less than 30 ml/min/1.73 m^2^ at the first visit. The data for underlying comorbidities were directly collected by the physician by reviewing patients’ electronic medical records on past medical history, prescription drugs, and lists of diagnoses (10th International Classification of Diseases [ICD-10]). The Charlson Comorbidity Index (CCI) was obtained through a previously reported equation using 17 disease categories defined by the ICD-10^23^.

### Statistical analysis

Chi-square tests for categorical variables were conducted to compare demographic details and comorbidity status. The normality of continuous variables was confirmed using histograms and the Shapiro–Wilk normality test. Most of the continuous variables were not normally distributed. The Student’s t-test was conducted for continuous variables of demographic and clinical data, including laboratory tests. Categorical variables are expressed as proportions (%) and continuous variables as mean ± standard deviation.

For comparison of the behavioral changes between before and during the COVID-19 pandemic, the Wilcoxon-signed rank test and McNemar’s test were used. Using the K-means cluster technique with the data of behavioral patterns before the COVID-19 pandemic and at the first visit, demographic details, and socio-economic status surveyed at the first visit, we divided the total study population into three clusters. Logistic regression analysis was conducted to identify the relative factors associated with behavioral changes and serum creatinine increment from the first to the second visit by adjusting for age, sex, education, and income status. The serum creatinine increment was defined as more than 1.5 times the serum creatinine level at the second visit compared with the first. Statistical analyses were performed using the R software (version 4.1.1. R Core Team [2021], R Foundation for Statistical Computing, Vienna, Austria. URL: https://www.R-project.org/). Statistical significance was set at *P* < 0.05.

### Ethical considerations

The Institutional Review Boards of the participating hospitals (Seoul National University Boramae Medical Center:10-2020-35; Keimyung University Dongsan Hospital:2020-04-056; Dongguk University Ilsan Hospital:2020-04-028; Hallym University Sacred Heart Hospital: HALLYM 2020-06-014; Ulsan University Hospital: UUH 2020-06-006) approved the study protocol and patient participation. This study was conducted following the principles of the Declaration of Helsinki. The participants provided written informed consent and participation was voluntary.

## Results

### Study population

**Figure 1:**
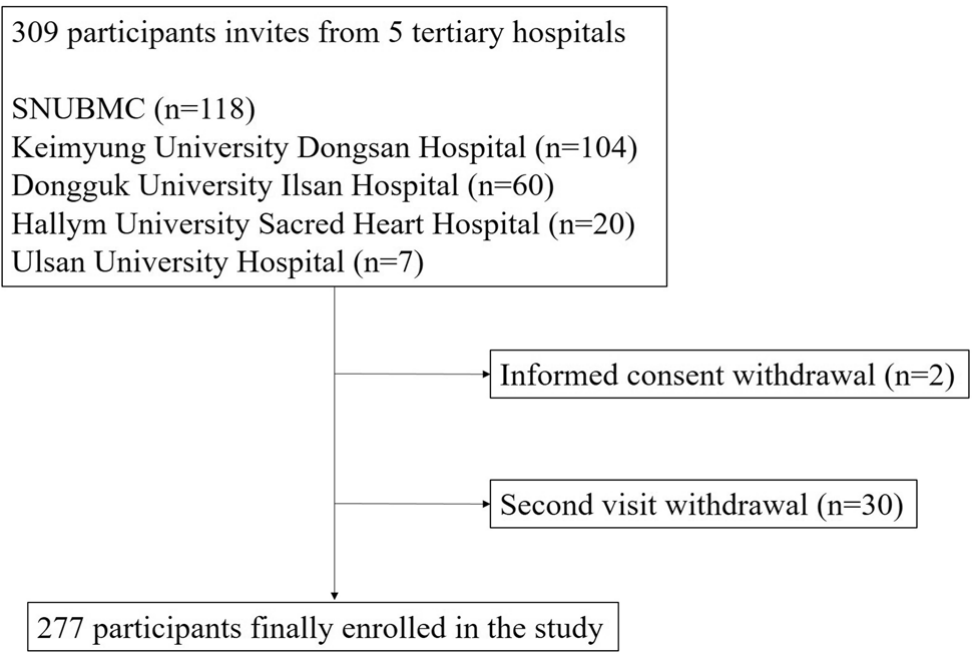
Diagram showing the study sample. SNUBMC, Seoul National University Boramae Medical Center.

Among the 309 patients selected through the inclusion and exclusion criteria, two who withdrew informed consent and 30 who did not complete the second visit were excluded. Therefore, 277 patients were included in the analysis (Fig. 1). The mean age of the sample was 61.11 ± 12.67 years (Table 1). Males and females constituted 66.8% and 33.2% of the total sample, respectively. Regarding education, those who had attained a level of middle school graduate or lower, high school graduate, and college graduate or higher accounted for 34.8%, 34.8%, and 30.4%, respectively. The proportion of patients with a monthly income of < 1 million won, 1–3 million won, and $\ge$ 3 million won was 31.1%, 29.0%, and 39.9%, respectively. Of the total sample, 45.1% showed high-risk perception. The mean serum creatinine, eGFR, and uPCR values were 1.83 ± 0.73 mg/dL, 43.03 ± 21.20 mL/min/1.73 m^2^, and 0.93 ± 1.49 g/g, respectively. A total of 124 (44.8%) patients showed strong behavioral changes. The only variable with a significant difference between the groups with and without strong behavioral changes was eGFR, which was 46.37 ± 22.86 mL/min/1.73 m^2^ and 40.31 ± 19.40 mL/min/1.73 m^2^, respectively (Table 1).

**Table 1:** Demographic and clinical characteristics of patients with chronic kidney disease at second visit.

Variables	Total population (n = 277)	Strong behavioral change (n = 124)	Non-strong behavioral change (n = 153)	p-value
Age, year	61.11 ± 12.67	60.46 ± 12.37	61.63 ± 12.92	0.442
Female (%)	33.2	35.5	31.4	0.552
Height, cm	164.5 ± 8.9	164.8 ± 9.3	164.2 ± 8.5	0.539
Body weight, kg	69.55 ± 13.33	70.24 ± 13.61	69.00 ± 13.13	0.444
BMI, kg/m^2^	25.62 ± 3.93	25.76 ± 4.08	25.50 ± 3.82	0.594
**Education (n = 276)**				0.028
Middle school or lower (%)	34.8	26.6	41.5	
High school (%)	34.8	41.1	29.7	
College or higher education (%)	30.4	32.3	28.8	
**Income (n = 276)**				0.001
< 1,000,000 won/month (%)	31.1	20.2	40.1	
1,000,000–3,000,000 won/month (%)	29.0	36.3	23.0	
$\ge$ 3,000,000 won/month (%)	39.9	43.5	36.9	
Risk perception ≥ 4 (%)	45.1	49.2	41.8	0.270
Good health perception (%)	52.7	50.0	54.9	0.489
Systolic blood pressure, mmHg (n = 275)	130.70 ± 15.08	131.50 ± 14.62	130.10 ± 15.48	0.434
Diastolic blood pressure, mmHg (n = 275)	75.06 ± 12.01	76.38 ± 11.60	73.97 ± 12.27	0.097
WBC, × 10^3^/µL (n = 275)	9.89 ± 47.56	7.35 ± 2.22	10.19 ± 50.28	0.380
Hemoglobin, g/dL (n = 275)	12.69 ± 2.03	12.52 ± 2.33	12.71 ± 2.00	0.676
Platelet, × 10^3^/µL (n = 275)	222.6 ± 60.8	233.1 ± 61.2	221.3 ± 60.8	0.336
Glucose, mg/dL (n = 271)	122.8 ± 48.58	115.9 ± 27.80	123.6 ± 50.48	0.211
Total cholesterol, mg/dL (n = 273)	157.0 ± 39.0	159.7 ± 46.6	156.7 ± 38.1	0.740
Protein, g/dL (n = 275)	6.99 ± 0.56	7.06 ± 0.53	6.98 ± 0.56	0.461
Albumin, g/dL (n = 275)	4.30 ± 0.39	4.32 ± 0.29	4.30 ± 0.40	0.725
Bilirubin, mg/dL (n = 272)	0.53 ± 0.29	0.55 ± 0.31	0.53 ± 0.29	0.646
Uric acid, mg/dL (n = 274)	6.37 ± 1.71	6.50 ± 2.32	6.35 ± 1.63	0.733
BUN, mg/dL (n = 276)	29.27 ± 13.03	27.22 ± 15.43	29.51 ± 12.73	0.446
Creatinine, mg/dL (n = 276)	1.83 ± 0.73	1.75 ± 0.79	1.90 ± 0.67	0.108
GFR(CKD-EPI), mL/min/1.73 m^2^ (n = 276)	43.03 ± 21.20	46.37 ± 22.86	40.31 ± 19.40	0.020
uPCR, g/g (n = 271)	0.93 ± 1.49	0.78 ± 1.15	1.06 ± 1.71	0.114

BUN, blood urea nitrogen; GFR, glomerular filtration rate; CKD-EPI, Chronic Kidney Disease Epidemiology Collaboration; uPCR, urine protein-to-creatinine ratio.

### Behavioral changes before and during the COVID-19 pandemic

In the total sample, the four physical distancing-related behavioral changes, excluding hours of private vehicle use, changed significantly toward reinforcing physical distancing during the patients’ first visit, compared to before the COVID-19 pandemic (Table 2, Fig. S1). The above three physical distancing-related behavioral changes remained intensified during the second visit, while hours of private vehicle use showed no statistically significant difference from before the COVID-19 pandemic to the second visit. The seven hygiene-related behavioral changes, excluding the number of clothes used before laundry, were significantly strengthened at the first visit compared to before the COVID-19 pandemic. Among the above six strengthened hygiene-related behavioral changes, all excluding the wearing of face masks weakened at the second visit compared to the first visit. Three of the five weakened hygiene-related behaviors (shower, laundry, and home cleaning) showed similar or further reduction compared to before the COVID-19 pandemic. A decrease in exercise frequency was interpreted as applying physical distancing. It showed a tendency to decrease and was significantly reduced at the second visit compared to before the COVID-19 pandemic.

**Table 2:** Variable changes before COVID-19 pandemic and at the first and second visit of the patients.

Variable changes	Before first visit	First visit	Second visit	p-value*	p-value^†^	p-value^‡^
Public transport use (numbers/week)	1.85 ± 2.82	1.33 ± 2.49	1.47 ± 2.79	< 0.001	0.005	0.499
Private vehicle use (hours/day)	1.08 ± 2.07	1.08 ± 2.03	1.03 ± 2.12	0.842	0.216	0.103
Public place visit (numbers/week)	2.31 ± 2.79	1.14 ± 2.31	1.30 ± 2.42	< 0.001	< 0.001	0.320
Staying at home (hours/day)	12.08 ± 5.80	13.73 ± 5.80	13.22 ± 6.01	< 0.001	< 0.001	0.287
Handwashing (times/day)	3.36 ± 3.55	5.98 ± 5.04	5.33 ± 3.98	< 0.001	< 0.001	< 0.001
Showering (times/day)	1.09 ± 0.68	1.30 ± 0.77	0.97 ± 0.59	< 0.001	0.010	< 0.001
Face mask use (times/week)	0.92 ± 2.09	6.26 ± 1.69	6.33 ± 1.75	< 0.001	< 0.001	0.596
Hand sanitizer use (numbers/week)	1.40 ± 5.15	8.63 ± 9.72	7.20 ± 10.03	< 0.001	< 0.001	< 0.001
Clothes used before laundry (numbers)	1.95 ± 1.11	2.04 ± 1.43	2.06 ± 2.07	0.442	0.765	0.586
Laundry (times/week)	3.23 ± 2.11	3.60 ± 2.14	3.41 ± 2.19	< 0.001	0.115	0.200
Home cleaning (times/week)	4.51 ± 2.50	4.98 ± 2.64	4.94 ± 3.27	< 0.001	0.063	0.447
Exercise frequency grade	2.68 ± 2.75	2.52 ± 2.67	2.29 ± 2.71	0.069	0.011	0.106
Regular exercise (%)		55.6	57.8			0.594
Creatinine, mg/dL		1.79 ± 0.65	1.83 ± 0.73			0.006
eGFR, mL/min/1.73 m^2^		43.35 ± 20.47	43.03 ± 21.20			0.380
uPCR, g/g		0.81 ± 1.27	0.93 ± 1.49			0.013

GFR, glomerular filtration rate; uPCR, urine protein-to-creatinine ratio.

*Comparison of data before the first visit with data from the first visit.

^†^Comparison of data from the second visit with data from before the first visit.

^‡^Comparison of data from the second visit with data from the first visit.

In participants with a high-risk perception, behavioral changes related to physical distancing showed the same trend as in the total sample (Table S1, Fig. S2). However, among them, the frequency of doing laundry and home cleaning decreased to pre-pandemic levels at the second visit. In contrast, in the remaining patients, the number of showers did not decrease compared to the pre-pandemic period, and the frequency of laundry and home cleaning was maintained, with no significant difference from the first visit (Fig. S3). However, in the non-high-risk perception group, the frequency of public transportation use tended to decrease from the first to the second visit. Overall, the exercise frequency decreased at the first visit compared to the pre-COVID-19 data in the high-risk perception group, but there was no significant difference in both the high and non-high-risk perception groups from the first to the second visit.

### Clustering analysis

Three cluster groups were divided using K-means clustering: cluster A (n = 57), cluster B (n = 187), and cluster C (n = 33) (Table 3). Cluster A had the oldest patients, a high number of females, and those with high-risk perception, while the numbers of those with high education and high income were low. Meanwhile, patients in cluster C showed the opposite tendency to those in cluster A. Those in cluster B showed characteristics that were a mix of those in clusters A and B. Patients in cluster C had the highest frequencies of private vehicle use and visits to public places, and the lowest time staying at home, at the first visit.

**Table 3:** Variable difference and behavioral characteristics at first visit among cluster subgroups.

Variable	Cluster A (n = 57)	Cluster B (n = 187)	Cluster C (n = 33)	p-value
Age	69.14 ± 8.33	59.13 ± 12.91	58.45 ± 12.19	< 0.001
Female (%)	45.6	31.6	21.2	0.042
BMI	25.25 ± 3.54	25.16 ± 4.01	26.87 ± 4.65	0.314
College or higher education (%)	8.8	34.8	42.4	< 0.001
High-income^a^ (%)	12.3	43.9	63.6	< 0.001
Good health perception (%)	40.4	57.2	48.5	0.072
High-risk perception (%)	59.6	43.4	30.3	0.018
Strong behavior change (%)	43.9	45.5	42.4	0.938
DM (%)	59.6	49.2	54.5	0.366
HTN (%)	82.5	79.7	90.9	0.303
Public transport use (numbers/week)	0.72 ± 1.32	1.54 ± 2.76	1.21 ± 2.29	0.352
Private vehicle use (hours/day)	0.82 ± 1.58	1.04 ± 2.11	1.75 ± 2.17	0.004
Public place visit (numbers/week)	0.95 ± 1.91	1.11 ± 2.43	1.61 ± 2.21	0.032
Staying at home (hours/day)	17.82 ± 4.69	12.72 ± 5.64	12.33 ± 5.41	< 0.001
Handwashing (times/day)	6.87 ± 6.46	5.74 ± 4.04	5.85 ± 7.02	0.022
Showering (times/day)	1.10 ± 0.55	1.38 ± 0.85	1.17 ± 0.54	0.018
Face mask use (times/week)	5.86 ± 2.16	6.34 ± 1.56	6.52 ± 1.42	0.335
Hand sanitizer use (numbers/week)	7.81 ± 9.19	8.87 ± 10.45	8.73 ± 5.59	0.656
Clothes used before laundry (numbers)	2.03 ± 1.38	2.05 ± 1.41	2.03 ± 1.63	0.688
Laundry (times/week)	3.77 ± 2.19	3.63 ± 2.18	3.15 ± 1.82	0.494
Home cleaning (times/week)	4.98 ± 2.51	5.10 ± 2.61	4.30 ± 2.99	0.196
Exercise frequency grade	2.90 ± 3.20	2.55 ± 2.59	1.70 ± 1.93	0.441
Regular exercise (%)	42.1	41.2	63.6	0.054
Creatinine, mg/dL	1.91 ± 0.62	1.77 ± 0.65	1.66 ± 0.68	0.192
eGFR, mL/min/1.73 m^2^	35.35 ± 15.05	44.41 ± 20.34	51.16 ± 25.09	< 0.001
uPCR, g/g	0.72 ± 1.18	0.78 ± 1.24	1.11 ± 1.51	0.334

BMI, body mass index; DM, diabetes mellitus; HTN, hypertension; GFR, glomerular filtration rate; uPCR, urine protein-to-creatinine ratio.

^a^Monthly average household income for 1 year ≥ 3 million Korean won or more.

In clusters A and B, the trend of physical distancing-related behavioral change was the same as that of the total sample (Figs. S4, 5, Table S2). However, in cluster C, the use of public transport did not decrease at the first visit compared with before COVID-19 (Fig. S6, Table S2). In addition, the restriction on visiting public places and the effort to stay at home were not maintained during at the second visit. There were no statistically significant differences in the two physical distancing-related behaviors between before the COVID-19 pandemic and the second visit.

Patients in clusters A and B showed reinforced behavior levels in six out of seven hygiene-related behaviors at the first visit, and weak behavior levels in four (handwashing, showering, using hand sanitizer, and laundry) and three (handwashing, showering, and using hand sanitizer) out of six hygiene-related behaviors, respectively, at the second visit. However, patients in cluster C showed behavioral reinforcement in five hygiene-related behaviors at the first visit and high levels of behavioral maintenance, except for home cleaning. In all clusters, the frequency of face mask use was strengthened at the first visit compared to before the COVID-19 pandemic and continued to be reinforced at the second visit.

### Factors associated with behavioral changes between the first and second visits

In 124 (44.8%) patients, more than six behaviors were strengthened or maintained at the second visit compared to the first visit. During the second visit period, the daily number of patients diagnosed with COVID-19 in Korea increased (Fig. S7). The mean number of confirmed COVID-19 cases in Korea from a week to a day before the patient’s second visit was calculated and categorized into three groups: ≤ 100 (n = 88, 31.8%), 100–180 (n = 93, 33.6%), and > 180 (n = 96, 34.7%). The number of daily confirmed COVID-19 cases within the week before the second visit was not associated with reinforcing behavior at the second visit (*P*-values 0.58 and 0.29) (Table 4). The high-risk perception for COVID-19 infection and perception of good health was also not associated with behavior maintenance or reinforcement at the second visit (*P* = 0.21 and 0.74, respectively). The cluster groups and high CCI, advanced CKD, and high uPCR also showed no significant association with strengthened or maintained behavioral changes. Patients with strong behavioral changes at baseline tended to strengthen or maintain six or more behaviors from the first to the second visit (adjusted odds ratio [OR] 1.99, 95% confidence interval [CI] 1.19–3.34; *P* < 0.01). The weakening of eight or more behaviors compared to pre-COVID-19 data was also statistically associated only with strong behavioral changes (adjusted OR 0.42, 95% CI 0.24–0.73; *P* < 0.01). Additional analysis was conducted by dividing all behavioral changes into physical distancing-related behaviors, hygiene practices, and exercise. Only patients with strong behavioral changes were associated with the reinforcement of two or more physical distancing behaviors (adjusted OR 1.75, 95% CI 1.05–2.93; *P* = 0.03). Patients in cluster C significantly reinforced three or more hygiene-related behaviors compared to those in cluster A (adjusted OR 3.73, 95% CI 1.49–10.13; *P* < 0.01). Less exercise was not associated with any of these factors.

**Table 4:** Behavioral changes at second visit from enrollment by risk perception, previous behavioral change characteristics and comorbidities.

	Overall behavioral change strengthened or maintained^a^		Overall behavioral change weaken or remained^b^		Physical distance strengthened or maintained^c^		Hygiene strengthened or maintained^d^		Exercise less or maintained^e^	
	OR (95% CI)	p-value	OR (95% CI)	p-value	OR (95% CI)	p-value	OR (95% CI)	p-value	OR (95% CI)	p-value
**COVID patients**^**f**^** ≤ 100 (ref)**										
100–180	0.84 (0.45–1.56)	0.582	1.29 (0.67–2.48)	0.448	0.78 (0.42–1.45)	0.441	0.63 (0.33–1.17)	0.147	1.27 (0.67–2.44)	0.469
> 180	0.71 (0.37–1.34)	0.289	1.40 (0.72–2.75)	0.326	0.67 (0.35–1.27)	0.217	0.57 (0.29–1.08)	0.087	1.34 (0.69–2.62)	0.396
Risk perception ≥ 4	1.39 (0.83–2.36)	0.214	0.74 (0.43–1.28)	0.285	1.24 (0.73–2.11)	0.419	1.06 (0.63–1.78)	0.831	1.27 (0.74–2.18)	0.384
Good health perception	1.09 (0.66–1.79)	0.744	0.86 (0.51–1.45)	0.574	1.17 (0.71–1.94)	0.533	0.93 (0.57–1.53)	0.778	1.01 (0.60–1.70)	0.964
Strong behavior change	1.99 (1.19–3.34)	0.009	0.42 (0.24–0.73)	0.002	1.75 (1.05–2.93)	0.033	1.52 (0.91–2.56)	0.113	1.24 (0.73–2.13)	0.421
Cluster (ref. A)*										
Cluster B	1.24 (0.68–2.30)	0.483	1.12 (0.60–2.12)	0.730	1.03 (0.57–1.89)	0.928	1.66 (0.92–3.04)	0.094	1.07 (0.58–2.06)	0.824
Cluster C	1.50 (0.63–3.58)	0.361	0.54 (0.19–1.42)	0.225	0.79 (0.32–1.89)	0.592	3.73 (1.49–10.13)	0.007	1.08 (0.43–2.69)	0.864
CCI ≥ 4	0.85 (0.50–1.42)	0.528	1.11 (0.65–1.93)	0.695	1.01 (0.60–1.70)	0.984	0.99 (0.59–1.67)	0.981	0.69 (0.40–1.18)	0.174
Advanced CKD	1.06 (0.61–1.84)	0.835	0.89 (0.49–1.56)	0.680	0.95 (0.54–1.64)	0.842	1.44 (0.83–2.53)	0.198	0.92 (0.51–1.62)	0.767
uPCR (> 1 g/g)	0.82 (0.44–1.53)	0.543	0.71 (0.48–1.76)	0.824	1.34 (0.72–2.48)	0.357	0.78 (0.42–1.43)	0.422	1.17 (0.62–2.18)	0.629

CCI, Charlson comorbidity index; CKD, chronic kidney disease; uPCR, urine protein-to-creatinine ratio.

^a^ ≥ 6 out of 13 all behaviors that became strengthened compared to before the COVID-19 pandemic and maintained or strengthened compared to the first visit.

^b^ ≥ 8 out of 12 all behaviors, except regular exercise, that became weaker or remained compared to before the COVID-19 pandemic.

^c^ ≥ 2 out of 4 physical distance-related behaviors that became strengthened compared to before the COVID-19 pandemic and maintained or strengthened compared to the first visit.

^d^ ≥ 3 out of 7 hygiene management behaviors that became strengthened compared to before the COVID-19 pandemic and maintained or strengthened compared to the first visit.

^e^Frequency of exercise became less compared to before the COVID-19 pandemic and was maintained or less compared to the first visit.

^f^Mean number of domestic COVID-19 confirmed patients per day from a week to a day before the second visit.

*Univariable logistic regression model without adjustment of age, sex, education and income.

### Factors associated with serum creatinine increment from the first visit

A total of 151 (54.5%) patients had increased serum creatinine levels at the second visit compared to the first visit. The number of daily confirmed COVID-19 cases within the week before the second visit, high-risk perception, good health perception, strong behavioral changes, cluster group, high CCI, and advanced CKD were not associated with serum creatinine increment (Table S3). Only uPCR > 1 g/g was associated with increased serum creatinine levels (adjusted OR 4.29, 95% CI 2.18–8.88; *P* < 0.01).

## Discussion

As there is no definitive antiviral treatment for COVID-19 infection to date, prevention is of utmost importance. Although COVID-19 vaccines have been developed, behavioral changes, such as physical distancing and wearing a mask, have been emphasized to prevent infection^2,4,5,20,24–30^. Among the 12 behaviors, including four physical distancing-related behaviors, seven hygiene practice behaviors, and frequency of exercise, nine behaviors were significantly reinforced at the first visit compared to before the COVID-19 pandemic. However, at the second visit, only four (use of public transport, visiting public places, duration of staying at home, and frequency of wearing face masks) of the above nine remained reinforced; the remaining five (all hygiene-related) behaviors weakened, except for wearing a face mask. The reason for the significant decrease in the frequency of the five hygiene-related behaviors at the second visit compared to before the COVID-19 pandemic may be that many patients’ second visit was during winter. However, follow-up and further studies are necessary to explore these reduced frequencies. Overall, behavioral changes to prevent COVID-19 infection in patients with CKD were reinforced at the beginning of the pandemic. However, after 3 months, many hygiene-related reinforced behaviors became weaker.

A previous study showed that risk perception was positively and significantly correlated with an index of preventive health behaviors, such as handwashing, wearing a face mask, and physical distancing^31^. In this study, patients with high-risk perception showed more reinforced behavioral changes at the first visit than before the COVID-19 pandemic. However, patients with high-risk perception maintained a lower number of reinforced hygiene-related behaviors at the time of the second visit compared to the non-high-risk perception group. Those with a high-risk perception of COVID-19 infection showed a strong initial behavioral change, but this tended not to be maintained 3 months later. Reinforced physical distancing-related behaviors were maintained at the second visit in both the high and non-high-risk perception groups. However, patients with high-risk perception showed more weakened hygiene-related behavioral changes compared to their counterparts. Therefore, even if patients with high-risk perception showed infection risk aversion behavioral changes during the onset of the COVID-19 pandemic, it is necessary to encourage them to further strengthen hygiene-related behaviors over time and monitor their maintenance of behavioral reinforcement^8,32^.

The patients included in this study may have had different lifestyles according to their socio-economic status and demographics, which affected their behavioral characteristics^33^. Furthermore, their behavioral characteristics have complex relationships. Therefore, given the limitations of using conventional statistical techniques, the patients were divided into groups with three characteristics through clustering, which is an unsupervised machine learning algorithm^34^. Cluster C was the youngest and had the highest educational level and income, shorter durations of staying at home, the longest time for using private vehicles, and the highest frequency of visiting public places. They had active social and economic lifestyles and practiced less physical distancing. On the contrary, cluster A did not actively engage in social and economic activities. Unlike cluster C, cluster A maintained physical distancing well after the COVID-19 pandemic, including at the time of their second visit. However, regarding hygiene-related behavior, cluster A did not maintain the behavioral reinforcement at the second visit compared to the first visit, while cluster C did. In particular, handwashing and the use of hand sanitizers, considered important for the prevention of COVID-19 infection, remained significantly higher during the second visit in cluster C^28,30^. Although cluster A had the highest risk perception, it showed weakened hygiene-related behavioral changes at the second visit. Cluster A spent more time at home than cluster C and visited public places less frequently, showing a less active social and economic lifestyle. Therefore, cluster A tended to maintain the overall physical distancing-related behavior strengthened even at the second visit, and the need for showering or handwashing might be less felt compared to cluster C. It is necessary to continuously provide close monitoring for hygiene practice, especially to recommend handwashing and hand sanitizer use, to patients who are older and not active in socio-economic activities, such as those in cluster A.

Only strong behavioral changes (≥ 5 out of 12 behaviors) at the first visit compared to before the COVID-19 pandemic were associated with behavioral reinforcement or maintenance at the second visit. These results imply that patients who showed active behavioral reinforcement in the early stages of the COVID-19 pandemic had a tendency to reinforce these behaviors even after 3 months. Strong behavioral changes were associated with the reinforcement of physical distancing-related behavior among all behaviors to prevent COVID-19 infection. Therefore, it is necessary to note the maintenance of behavioral changes in the population that did not show strong behavioral changes after the initiation of the COVID-19 pandemic.

Maintenance and reduction of exercise at the second visit were not associated with any of these factors. In this study, a decrease in exercise frequency was interpreted as a behavioral change related to physical distancing and regarded as a risk aversion behavior. Exercise frequency decreased after the COVID-19 pandemic, which could be because the patients refrained from outdoor exercise and fitness clubs had to be closed owing to the national policy. However, whether lowering exercise frequency can help reduce the incidence of COVID-19 by reducing exposure risk during the pandemic may depend on the type of exercise, location, and comorbidities. A previous study showed that physical inactivity is strongly associated with an increased risk of severe COVID-19 outcomes^35^. Further studies are necessary to identify whether only outdoor exercise decreased or whether this was also true for exercise at home.

Serum creatinine and proteinuria levels were significantly increased between the first and second visits. This might have been caused by decreased adherence to treatment and loss of follow- up visits during the pandemic. The number of COVID-19 cases, level of risk perception, level of good health perception, the intensity of behavioral change, cluster, the severity of comorbidity, and CKD stage were not related to serum creatinine increase during the 3-month follow-up period. High proteinuria was the only factor associated with the risk of increased serum creatinine levels over 3 months. Previous studies have also reported that proteinuria is a biomarker for predicting renal deterioration^36,37^. However, the follow-up period of 3 months was short, making it difficult to evaluate renal function deterioration and other clinical outcomes, including major adverse cardiovascular events, COVID-19 infection, and mortality.

This study has several limitations. First, the sample size was small. Second, since this cohort consisted only of patients with CKD, the results cannot be compared with those of the general population without CKD. Third, in this study, a reduction in exercise frequency was interpreted as a behavioral change indicating a propensity for implementing physical distancing. However, exercising at home and in fitness clubs or public places with many people was not considered separately. Finally, the 3-month follow-up period was too short to identify significant factors affecting behavioral changes, and the seasonal effect on behavioral changes during the study period was not sufficiently considered.

During the COVID-19 pandemic, behavioral changes in patients with CKD were more prominent in the initial stages, but they weakened after 3 months. Even if the initial COVID-19 infection risk perception was high, behavioral changes were not sustained over time, and people who showed more active behavioral changes at the beginning had a higher tendency to maintain reinforced behavior over time. In addition, young, highly educated, and high-income patients with an active socio-economic lifestyle did not practice physical distancing but had strong hygiene practices, while their counterparts showed the opposite behavioral change pattern. Continuous education and monitoring must be emphasized in patients with CKD who cannot adequately maintain intensified behavioral changes. Further research on COVID-19 risk aversion behavior and its changes over time on long-term clinical outcomes is highly recommended.

## Supplementary Information

Supplementary Information.

## Data Availability

Drs. J Lee and JP Lee had full access to all study data and take responsibility for the integrity of the data and the accuracy of the analysis. Data from this study can be provided after anonymizing personal information at the request of the researcher.

## References

[1] Hartley DM, Perencevich EN (2020). Public health interventions for COVID-19: Emerging evidence and implications for an evolving public health crisis. JAMA.

[2] Chu DK (2020). Physical distancing, face masks, and eye protection to prevent person-to-person transmission of SARS-CoV-2 and COVID-19: A systematic review and meta-analysis. Lancet.

[3] Davies NG (2020). Effects of non-pharmaceutical interventions on COVID-19 cases, deaths, and demand for hospital services in the UK: A modelling study. Lancet Public Health.

[4] Masters NB (2020). Social distancing in response to the novel coronavirus (COVID-19) in the United States. PLoS ONE.

[5] Shahnazi H (2020). Assessing preventive health behaviors from COVID-19: A cross sectional study with health belief model in Golestan province, northern of Iran. Infect. Dis. Poverty.

[6] Kim S, Ko Y, Kim YJ, Jung E (2020). The impact of social distancing and public behavior changes on COVID-19 transmission dynamics in the Republic of Korea. PLoS ONE.

[7] Knell G, Robertson MC, Dooley EE, Burford K, Mendez KS (2020). Health behavior changes during COVID-19 pandemic and subsequent “Stay-at-Home” orders. Int. J. Environ. Res. Public Health.

[8] Michie S, West R (2021). Sustained behavior change is key to preventing and tackling future pandemics. Nat. Med..

[9] Ning L (2020). The impacts of knowledge, risk perception, emotion and information on citizens’ protective behaviors during the outbreak of COVID-19: A cross-sectional study in China. BMC Public Health.

[10] Porat T, Nyrup R, Calvo RA, Paudyal P, Ford E (2020). Public health and risk communication during COVID-19—enhancing psychological needs to promote sustainable behavior change. Front. Public Health.

[11] Flaxman S (2020). Estimating the effects of non-pharmaceutical interventions on COVID-19 in Europe. Nature.

[12] Lai S (2020). Effect of non-pharmaceutical interventions to contain COVID-19 in China. Nature.

[13] Noor AU, Maqbool F, Bhatti ZA, Khan AU (2020). Epidemiology of CoViD-19 pandemic: Recovery and mortality ratio around the globe. Pak. J. Med. Sci..

[14] Cai R (2021). Mortality in chronic kidney disease patients with COVID-19: A systematic review and meta-analysis. Int. Urol. Nephrol..

[15] Ozturk S (2020). Mortality analysis of COVID-19 infection in chronic kidney disease, haemodialysis and renal transplant patients compared with patients without kidney disease: A nationwide analysis from Turkey. Nephrol. Dial Transplant..

[16] Pakhchanian H (2021). Outcomes of COVID-19 in CKD patients: A multicenter electronic medical record cohort study. Clin. J. Am. Soc. Nephrol..

[17] Yanez ND, Weiss NS, Romand JA, Treggiari MM (2020). COVID-19 mortality risk for older men and women. BMC Public Health.

[18] Jdiaa SS (2022). COVID-19 and chronic kidney disease: An updated overview of reviews. J. Nephrol..

[19] Chan ASW, Ho JMC, Li JSF, Tam HL, Tang PMK (2021). Impacts of COVID-19 pandemic on psychological well-being of older chronic kidney disease patients. Front. Med..

[20] Kim EA (2020). Social distancing and public health guidelines at workplaces in Korea: Responses to coronavirus disease-19. Saf. Health Work.

[21] Jarvis CI (2020). Quantifying the impact of physical distance measures on the transmission of COVID-19 in the UK. BMC Med..

[22] McCormack GR, Doyle-Baker PK, Petersen JA, Ghoneim D (2021). Perceived anxiety and physical activity behaviour changes during the early stages of COVID-19 restrictions in community-dwelling adults in Canada: A cross-sectional study. BMJ Open.

[23] Quan H (2005). Coding algorithms for defining comorbidities in ICD-9-CM and ICD-10 administrative data. Med. Care.

[24] Baden LR (2021). Efficacy and safety of the mRNA-1273 SARS-CoV-2 vaccine. N. Engl. J. Med..

[25] Polack FP (2020). Safety and efficacy of the BNT162b2 mRNA COVID-19 vaccine. N. Engl. J. Med.

[26] Yousuf H (2020). Association of a public health campaign about coronavirus disease 2019 promoted by news media and a social influencer with self-reported personal hygiene and physical distancing in the Netherlands. JAMA Netw. Open.

[27] Esposito S, Principi N, Leung CC, Migliori GB (2020). Universal use of face masks for success against COVID-19: Evidence and implications for prevention policies. Eur. Respir. J..

[28] Haque M (2020). Handwashing in averting infectious diseases: Relevance to COVID-19. J. Popul. Ther. Clin. Pharmacol..

[29] Matuschek C (2020). Face masks: Benefits and risks during the COVID-19 crisis. Eur. J. Med. Res..

[30] Roshan R, Feroz AS, Rafique Z, Virani N (2020). Rigorous hand hygiene practices among health care workers reduce hospital-associated infections during the COVID-19 pandemic. J. Prim. Care Community Health.

[31] Dryhurst S (2020). Risk perceptions of COVID-19 around the world. J. Risk Res..

[32] Allegrante JP, Auld ME, Natarajan S (2020). Preventing COVID-19 and its sequela: “There is no magic bullet… it’s just behaviors”. Am. J. Prev. Med..

[33] Papageorge NW (2021). Socio-demographic factors associated with self-protecting behavior during the Covid-19 pandemic. J. Popul. Econ..

[34] Hartigan JA, Wong MA (1979). Algorithm AS 136: A k-means clustering algorithm. J. R. Stat. Soc. Ser. C Appl. Stat..

[35] Sallis R (2021). Physical inactivity is associated with a higher risk for severe COVID-19 outcomes: A study in 48 440 adult patients. Br. J. Sports Med..

[36] Abbate M, Zoja C, Remuzzi G (2006). How does proteinuria cause progressive renal damage?. J. Am. Soc. Nephrol..

[37] Zandi-Nejad K, Eddy AA, Glassock RJ, Brenner BM (2004). Why is proteinuria an ominous biomarker of progressive kidney disease?. Kidney Int. Suppl..

