# Supplementary material for: Longitudinal behavioral changes and factors related to reinforced risk aversion behavior among patients with chronic kidney disease during the COVID-19 pandemic
Source: Sci Rep. 2022 Sep 22;12:15780. doi: 10.1038/s41598-022-19787-0 (PMC9493162; doi:10.1038/s41598-022-19787-0)

|  | Non-high-risk perception (n=152) | | | | | | High-risk perception (n=125) | | | | | |
| --- | --- | --- | --- | --- | --- | --- | --- | --- | --- | --- | --- | --- |
| Variable changes | Before first visit | First visit | Second visit | p-value* | p-value^†^ | p-value^‡^ | Before first visit | First visit | Second visit | p-value* | p-value^†^ | p-value^‡^ |
| Public transport use (numbers/week) | 1.44±2.67 | 1.12±2.40 | 1.31±2.75 | <0.001 | 0.153 | 0.499 | 2.34±2.93 | 1.59±2.58 | 1.67±2.85 | <0.001 | 0.014 | 0.781 |
| Private vehicle use (hours/day) | 1.29±2.09 | 1.31±2.10 | 1.34±2.58 | 0.684 | 0.325 | 0.200 | 0.82±2.03 | 0.81±1.91 | 0.65±1.28 | 0.874 | 0.453 | 0.340 |
| Public place visit (numbers/week) | 2.26±3.12 | 1.24±2.60 | 1.57±2.69 | <0.001 | 0.002 | 0.080 | 2.37±2.34 | 1.01±1.89 | 0.97±2.02 | <0.001 | <0.001 | 0.672 |
| Staying at home (hours/day) | 12.74±5.38 | 14.26±5.69 | 13.76±5.92 | <0.001 | 0.009 | 0.514 | 11.29±6.20 | 13.08±5.89 | 12.57±6.17 | <0.001 | 0.020 | 0.385 |
| Handwashing (times/day) | 3.51±4.49 | 5.97±5.38 | 5.45±4.90 | <0.001 | <0.001 | 0.005 | 3.18±1.86 | 6.00±4.61 | 5.18±2.45 | <0.001 | <0.001 | 0.013 |
| Showering (times/day) | 1.11±0.48 | 1.31±0.63 | 1.04±0.54 | <0.001 | 0.318 | <0.001 | 1.07±0.87 | 1.28±0.92 | 0.88±0.63 | <0.001 | 0.014 | <0.001 |
| Face mask use (times/week) | 0.91±2.11 | 6.17±1.72 | 6.30±1.77 | <0.001 | <0.001 | 0.408 | 0.92±2.08 | 6.38±1.66 | 6.36±1.73 | <0.001 | <0.001 | 0.918 |
| Hand sanitizer use (numbers/week) | 1.96±6.56 | 9.45±11.10 | 7.36±9.54 | <0.001 | <0.001 | <0.001 | 0.72±2.39 | 7.63±7.65 | 7.00±10.62 | <0.001 | <0.001 | 0.024 |
| Clothes used before laundry (numbers) | 1.99±1.15 | 2.03±1.65 | 2.27±2.66 | 0.122 | 0.484 | 0.205 | 1.89±1.05 | 2.06±1.11 | 1.80±0.89 | 0.002 | 0.682 | 0.021 |
| Laundry (times/week) | 3.11±2.29 | 3.43±2.26 | 3.48±2.30 | 0.001 | 0.057 | 0.768 | 3.38±1.86 | 3.81±1.97 | 3.34±2.06 | <0.001 | 0.881 | 0.017 |
| Home cleaning (times/week) | 4.22±2.51 | 4.78±2.86 | 5.16±3.81 | <0.001 | 0.002 | 0.432 | 4.86±2.44 | 5.23±2.33 | 4.67±2.46 | <0.001 | 0.463 | 0.036 |
| Exercise frequency grade | 2.21±2.44 | 2.29±2.45 | 2.09±2.56 | 0.787 | 0.751 | 0.469 | 3.24±3.00 | 2.80±2.90 | 2.53±2.88 | 0.025 | 0.002 | 0.127 |
| Regular exercise (%) |  | 53.3 | 57.9 |  |  | 0.427 |  | 58.4 | 57.6 |  |  | >0.999 |
| Creatinine |  | 1.75±0.65 | 1.79±0.72 |  |  | 0.061 |  | 1.83±0.64 | 1.88±0.74 |  |  | 0.035 |
| eGFR |  | 45.50±20.99 | 45.06±21.53 |  |  | 0.394 |  | 40.74±19.58 | 40.54±20.61 |  |  | 0.755 |
| uPCR |  | 0.93±1.41 | 1.10±1.78 |  |  | 0.030 |  | 0.66±1.05 | 0.73±1.00 |  |  | 0.229 |

Table S1. Variable changes before COVID-19 pandemic, at first, and second visit in participants with non-high-risk perception and high-risk perception

a) Comparing variable changes at second visit between low risk and high-risk perception patients after age adjustment, sex, education, income, and mean number of domestic

*Comparing before first visit with first visit

†Comparing second visit with before first visit

^‡^ Comparing second visit with first visit

Abbreviations: GFR, glomerular filtration rate; uPCR, urine protein to creatinine ratio.

|  | Cluster A (n=57) | | | | | | Cluster B (n=187) | | | | | |
| --- | --- | --- | --- | --- | --- | --- | --- | --- | --- | --- | --- | --- |
| Variable changes | Before first visit | First visit | Second visit | p-value* | p-value^†^ | p-value^‡^ | Before first visit | First visit | Second visit | p-value* | p-value^†^ | p-value^‡^ |
| Public transport use (numbers/week) | 1.51±1.32 | 0.72±1.32 | 0.81±1.36 | <0.001 | 0.032 | 0.375 | 2.01±3.03 | 1.54±2.76 | 1.67±2.93 | <0.001 | 0.037 | 0.753 |
| Private vehicle use (hours/day) | 0.68±1.72 | 0.82±1.58 | 0.54±1.12 | 0.124 | 0.507 | 0.102 | 1.07±2.12 | 1.04±2.11 | 1.07±2.33 | 0.552 | 0.491 | 0.549 |
| Public place visit (numbers/week) | 2.05±2.50 | 0.95±1.91 | 0.47±1.04 | <0.001 | <0.001 | 0.120 | 2.36±2.50 | 1.11±2.43 | 1.39±2.61 | <0.001 | <0.001 | 0.158 |
| Staying at home (hours/day) | 15.40±5.89 | 17.82±4.69 | 16.88±5.70 | <0.001 | 0.044 | 0.246 | 11.22±5.49 | 12.72±5.64 | 12.41±5.84 | <0.001 | 0.005 | 0.795 |
| Handwashing (times/day) | 3.02±1.76 | 6.87±6.46 | 5.00±2.43 | <0.001 | <0.001 | 0.002 | 3.47±3.11 | 5.74±4.04 | 5.40±4.35 | <0.001 | <0.001 | 0.003 |
| Showering (times/day) | 0.89±0.47 | 1.10±0.55 | 0.85±0.56 | 0.003 | 0.617 | 0.008 | 1.17±0.77 | 1.38±0.85 | 0.99±0.61 | <0.001 | 0.005 | <0.001 |
| Face mask use (times/week) | 0.44±1.15 | 5.86±2.16 | 5.63±2.54 | <0.001 | <0.001 | 0.522 | 0.98±2.18 | 6.34±1.56 | 6.46±1.50 | <0.001 | <0.001 | 0.450 |
| Hand sanitizer use (numbers/week) | 0.53±1.54 | 7.81±9.19 | 6.63±10.32 | <0.001 | <0.001 | 0.025 | 1.63±5.93 | 8.87±10.45 | 7.28±10.51 | <0.001 | <0.001 | <0.001 |
| Clothes used before laundry (numbers) | 2.00±1.39 | 2.03±1.38 | 2.12±1.55 | 0.492 | >0.999 | 0.883 | 1.92±1.03 | 2.05±1.41 | 2.01±2.25 | 0.162 | 0.953 | 0.247 |
| Laundry (times/week) | 3.56±2.21 | 3.77±2.19 | 2.84±1.73 | 0.041 | 0.009 | <0.001 | 3.21±2.11 | 3.63±2.18 | 3.52±2.28 | <0.001 | 0.071 | 0.560 |
| Home cleaning (times/week) | 4.70±2.58 | 4.98±2.51 | 4.37±2.45 | 0.005 | 0.340 | 0.121 | 4.58±2.45 | 5.10±2.61 | 5.05±2.77 | <0.001 | 0.027 | 0.857 |
| Exercise frequency grade | 2.74±3.19 | 2.90±3.20 | 2.05±2.69 | 0.593 | 0.120 | 0.035 | 2.79±2.68 | 2.55±2.59 | 2.40±2.78 | 0.069 | 0.055 | 0.288 |
| Regular exercise (%) |  | 57.9 | 57.9 |  |  | >0.999 |  | 58.8 | 57.8 |  |  | >0.999 |
| Creatinine |  | 1.77±0.62 | 2.02±0.74 |  |  | 0.010 |  | 1.77±0.65 | 1.81±0.73 |  |  | 0.059 |
| eGFR |  | 35.35±15.05 | 34.06±15.66 |  |  | 0.115 |  | 44.41±20.34 | 39.20±21.23 |  |  | 0.798 |
| uPCR |  | 0.72±1.18 | 0.72±0.97 |  |  | 0.887 |  | 0.78±1.24 | 0.94±1.54 |  |  | 0.009 |

Table S2. Variable changes before COVID-19 pandemic, at first, and second visit among cluster subgroups

*Comparing before first visit with first visit

†Comparing second visit with before first visit

^‡^ Comparing second visit with first visit

Abbreviations: GFR, glomerular filtration rate; uPCR, urine protein to creatinine ratio.

|  | Cluster C (n=33) | | | | | |
| --- | --- | --- | --- | --- | --- | --- |
| Variable changes | Before first visit | First visit | Second visit | p-value* | p-value^†^ | p-value^‡^ |
| Public transport use (numbers/week) | 1.49±2.62 | 1.21±2.29 | 1.52±3.64 | 0.106 | 0.781 | 0.720 |
| Private vehicle use (hours/day) | 1.81±2.19 | 1.75±2.17 | 1.62±2.06 | 0.850 | 0.254 | 0.286 |
| Public place visit (numbers/week) | 2.46±2.27 | 1.61±2.21 | 2.21±2.65 | 0.004 | 0.483 | 0.341 |
| Staying at home (hours/day) | 11.21±5.45 | 12.33±5.41 | 11.52±5.54 | 0.009 | 0.683 | 0.153 |
| Handwashing (times/day) | 3.35±6.86 | 5.85±7.02 | 5.50±4.00 | <0.001 | <0.001 | 0.683 |
| Showering (times/day) | 1.02±0.32 | 1.17±0.54 | 1.03±0.45 | 0.089 | 0.963 | 0.127 |
| Face mask use (times/week) | 1.36±2.66 | 6.52±1.42 | 6.79±0.93 | <0.001 | <0.001 | 0.396 |
| Hand sanitizer use (numbers/week) | 1.61±4.26 | 8.73±5.59 | 7.70±6.15 | <0.001 | <0.001 | 0.321 |
| Clothes used before laundry (numbers) | 1.88±1.02 | 2.03±1.63 | 2.24±1.84 | 0.943 | 0.340 | 0.300 |
| Laundry (times/week) | 2.74±1.84 | 3.15±1.82 | 3.82±2.28 | 0.003 | 0.012 | 0.080 |
| Home cleaning (times/week) | 3.76±2.55 | 4.30±2.99 | 5.30±6.03 | 0.013 | 0.198 | 0.719 |
| Exercise frequency grade | 1.94±2.21 | 1.70±1.93 | 2.06±2.36 | 0.368 | 0.987 | 0.354 |
| Regular exercise (%) |  | 33.3 | 57.6 |  |  | 0.096 |
| Creatinine |  | 1.66±0.68 | 1.65±0.64 |  |  | 0.744 |
| eGFR |  | 51.16±25.09 | 51.07±24.62 |  |  | 0.906 |
| uPCR |  | 1.11±1.51 | 1.30±1.85 |  |  | 0.185 |

*Comparing before first visit with first visit

†Comparing second visit with before first visit

^‡^ Comparing second visit with first visit

Abbreviations: GFR, glomerular filtration rate; uPCR, urine protein to creatinine ratio.

Table S3. Creatinine elevation by risk perception, previous behavioral change characteristics and comorbidities after age adjustment, sex, education, income

|  | Creatinine increment comparing 1^st^ visit | |
| --- | --- | --- |
|  | OR (95% CI) | p-value |
| COVID patients^a)^ ≤ 100 (Ref) |  |  |
| 100 < COVID patients ≤ 180 | 0.61 (0.32-1.14) | 0.126 |
| 180 < COVID patients | 0.58 (0.30-1.10) | 0.099 |
| Risk perception ≥4 | 0.79 (0.46-1.32) | 0.368 |
| Good health perception | 1.03 (0.63-1.70) | 0.894 |
| Strong behavior change | 1.38 (0.83-2.33) | 0.217 |
| Cluster (Ref. A)^*^ |  |  |
| Cluster B | 0.77 (0.42-1.40) | 0.391 |
| Cluster C | 1.07 (0.45-2.61) | 0.876 |
| CCI ≥4 | 1.62 (0.96-2.73) | 0.072 |
| Advanced CKD | 0.98 (0.57-1.70) | 0.944 |
| uPCR (>1g/g) | **4.29 (2.18-8.88)** | **<0.001** |

a) Mean number of domestic COVID-19 confirmed patients per day from one day to one week before second visit

* Univariable logistic regression model, without adjustment of age, sex, education and income

Abbreviations: CCI, charlson comorbidity index; CKD, chronic kidney disease; uPCR, urine protein to creatinine ratio.

Figure S1. Diagram of variable changes before COVID-19 pandemic, at first, and second visit in total participants


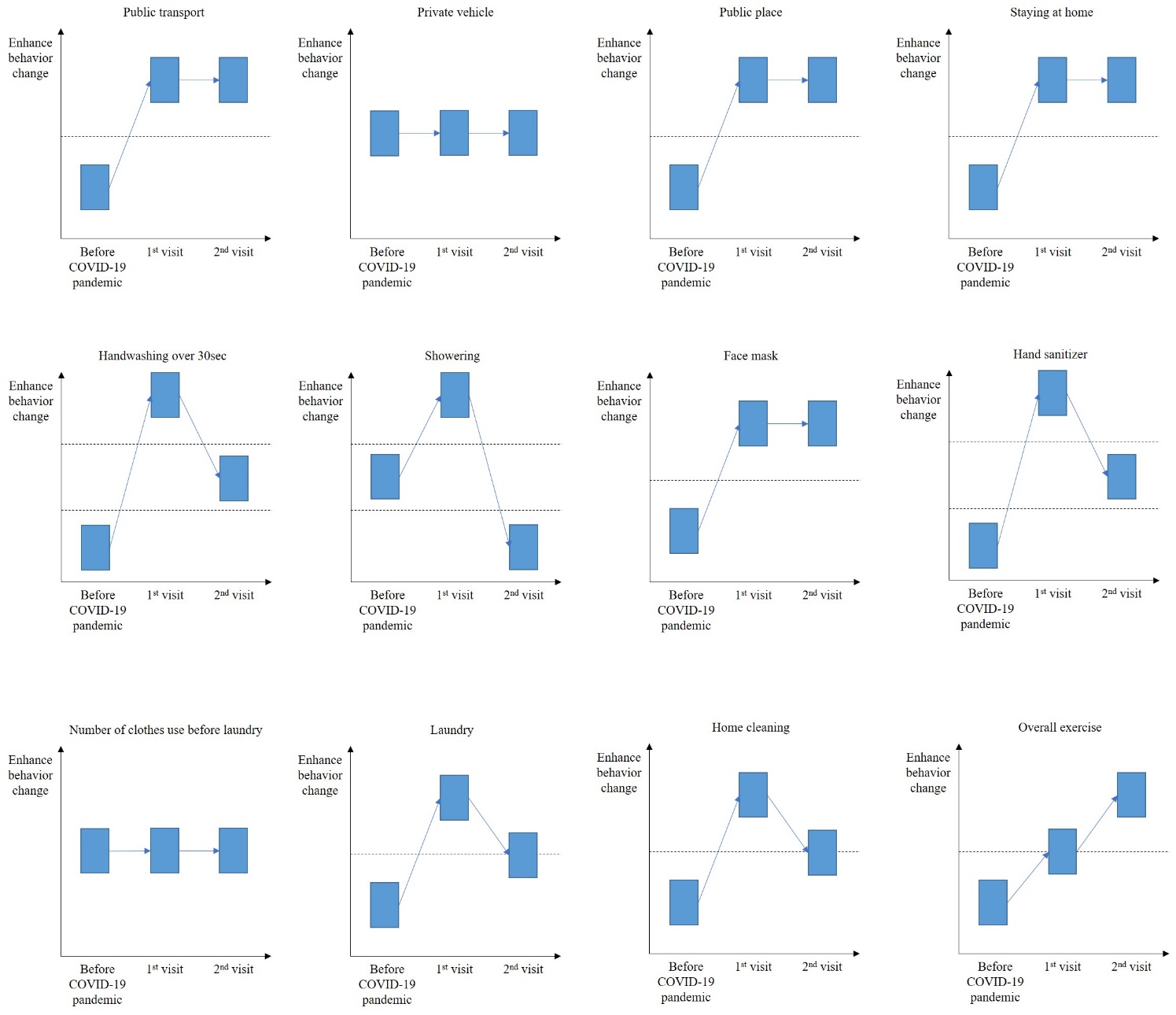


Figure S2. Diagram of variable changes before COVID-19 pandemic, at first, and second visit in participants with high-risk perception


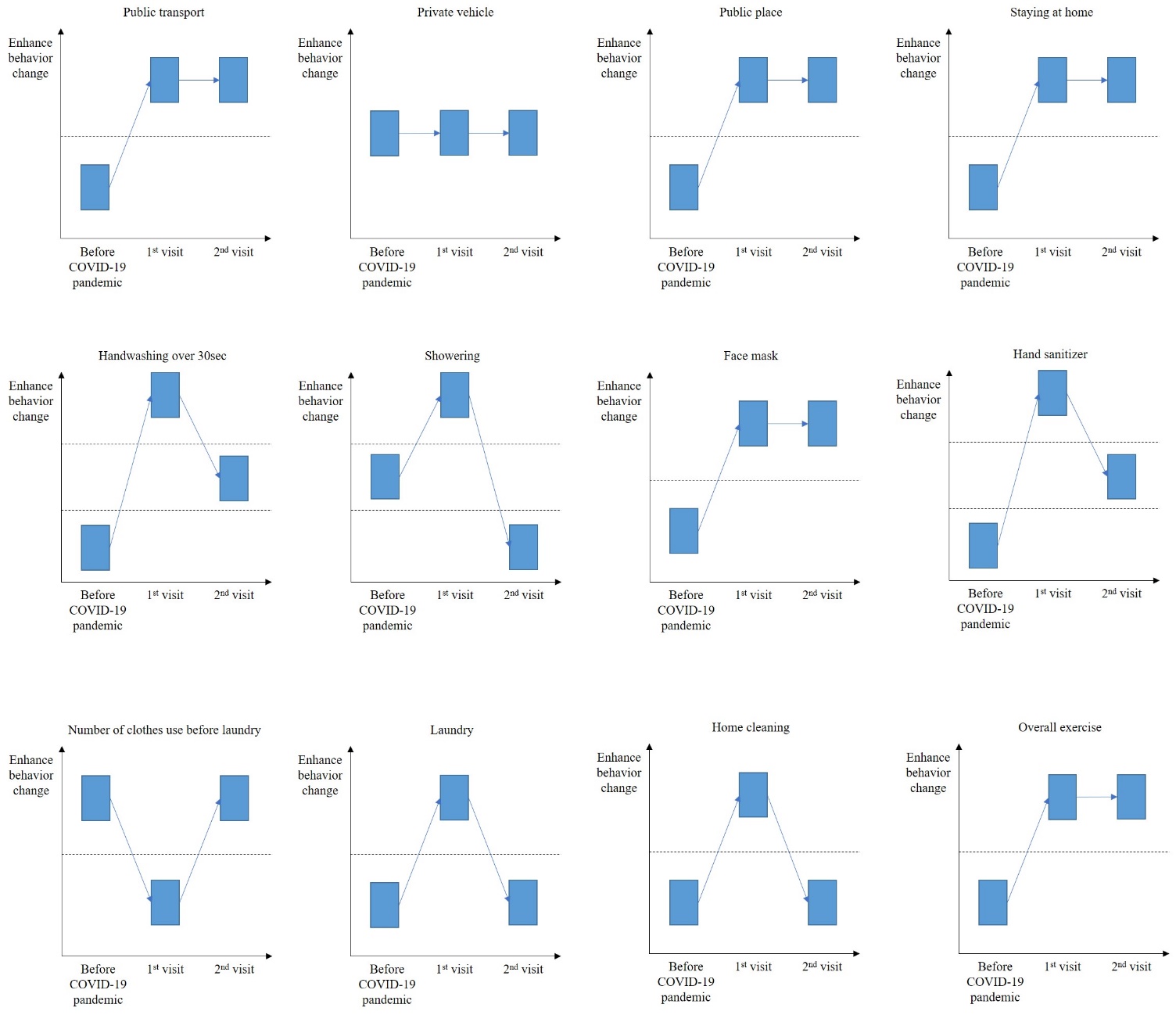


Figure S3. Diagram of variable changes before COVID-19 pandemic, at first, and second visit in participants with non-high-risk perception


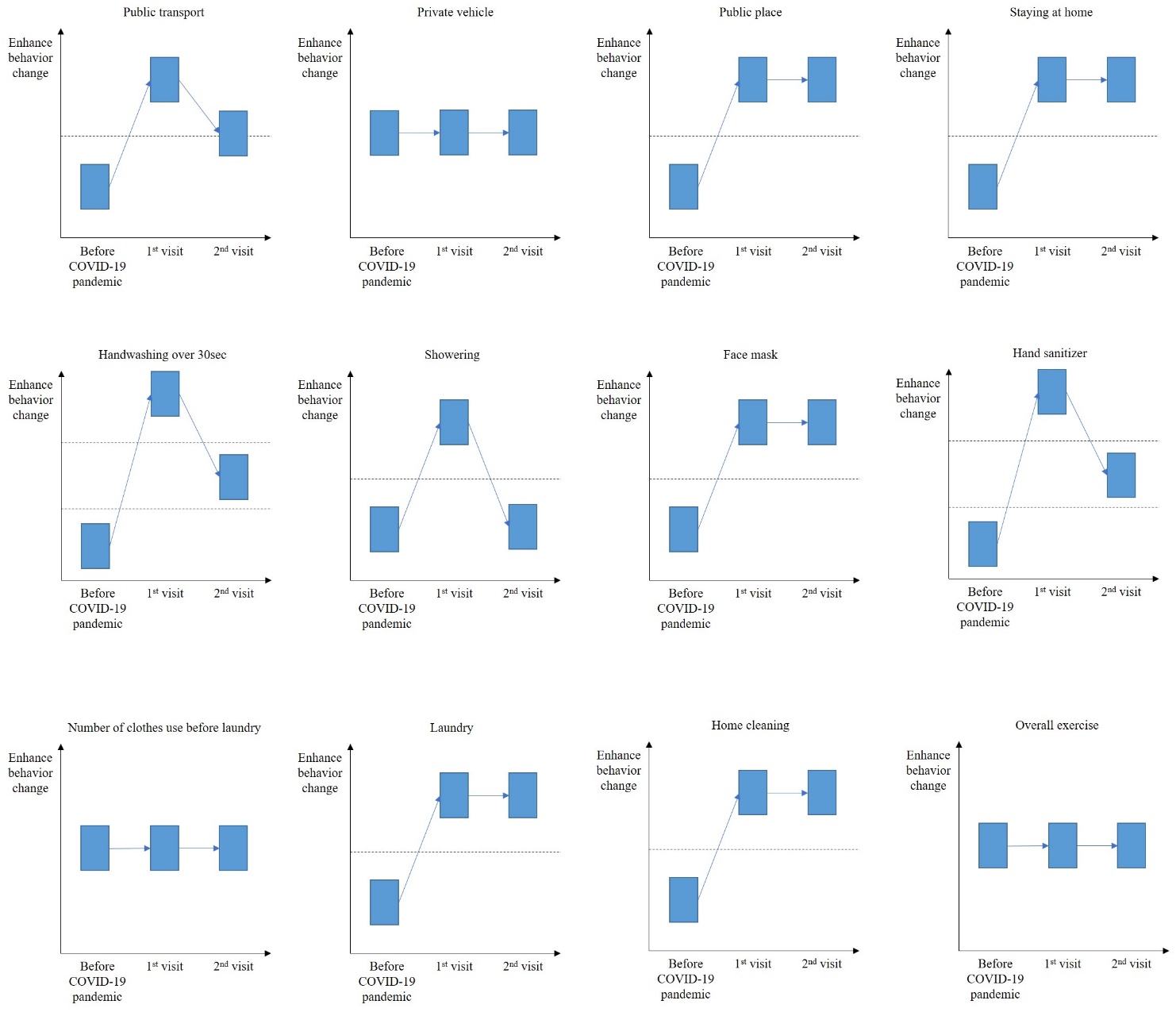


Figure S4. Diagram of variable changes before COVID-19 pandemic, at first, and second visit in cluster A subgroup


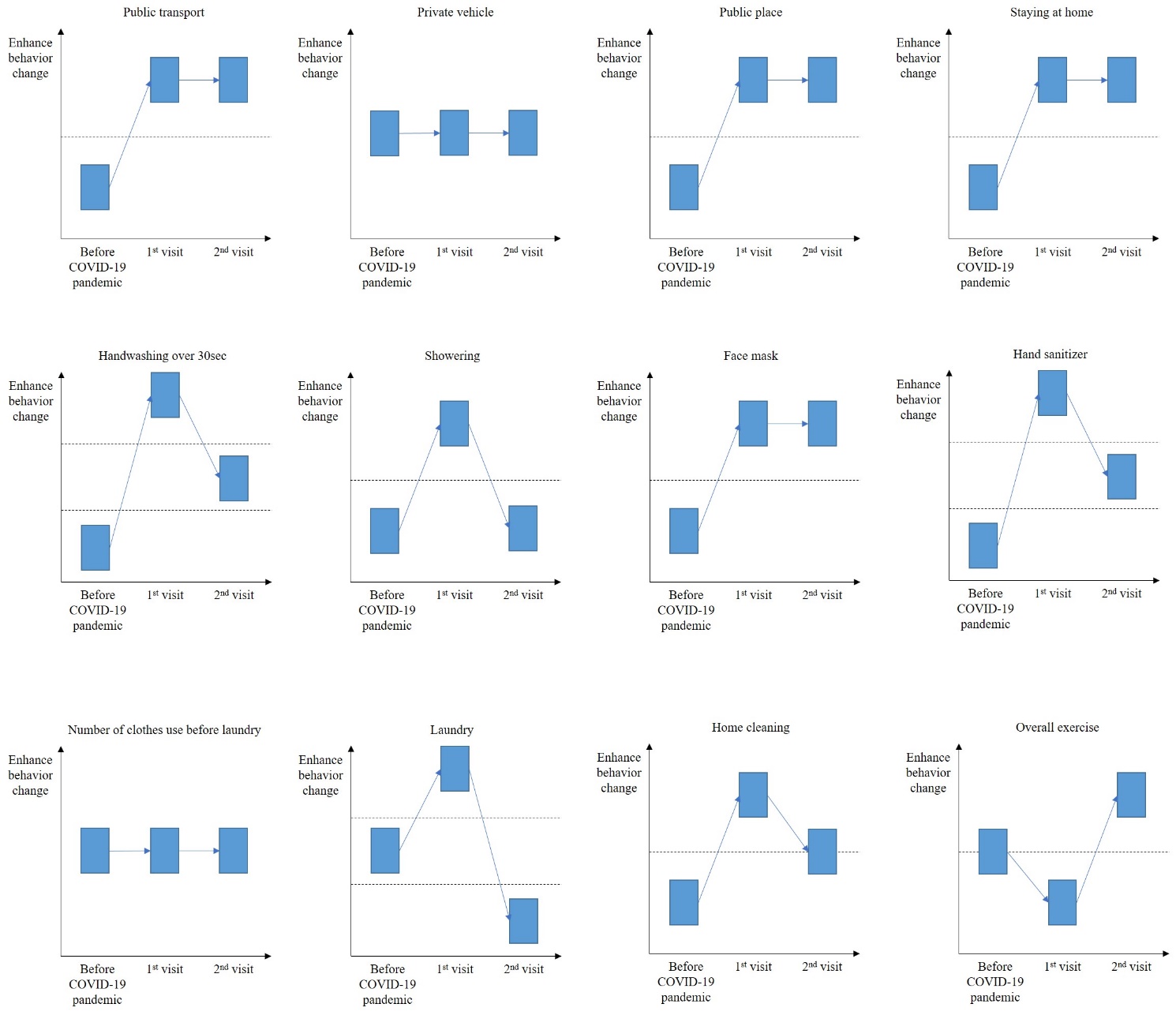


Figure S5. Diagram of variable changes before COVID-19 pandemic, at first, and second visit in cluster B subgroup


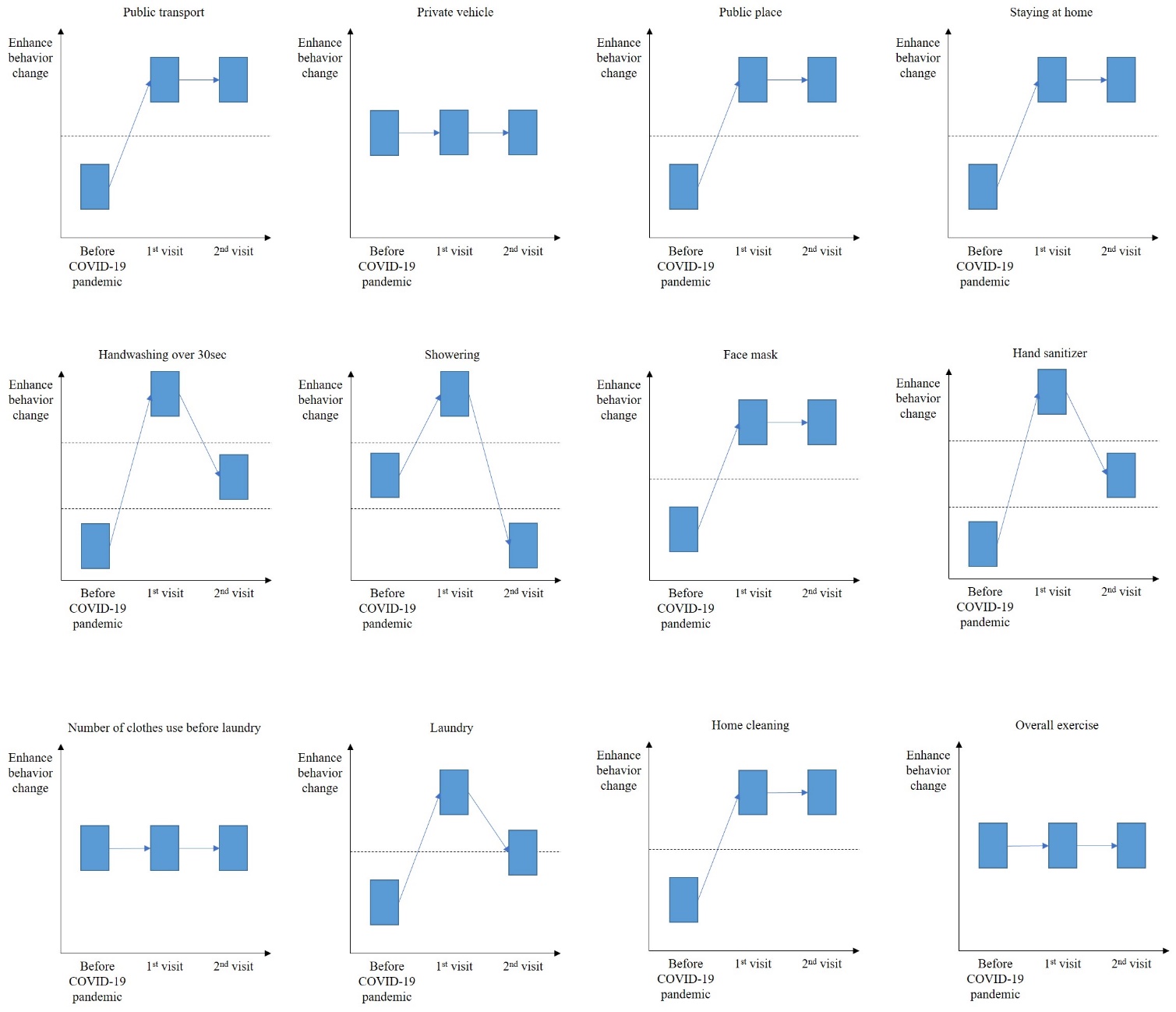


Figure S6. Diagram of variable changes before COVID-19 pandemic, at first, and second visit in cluster C subgroup


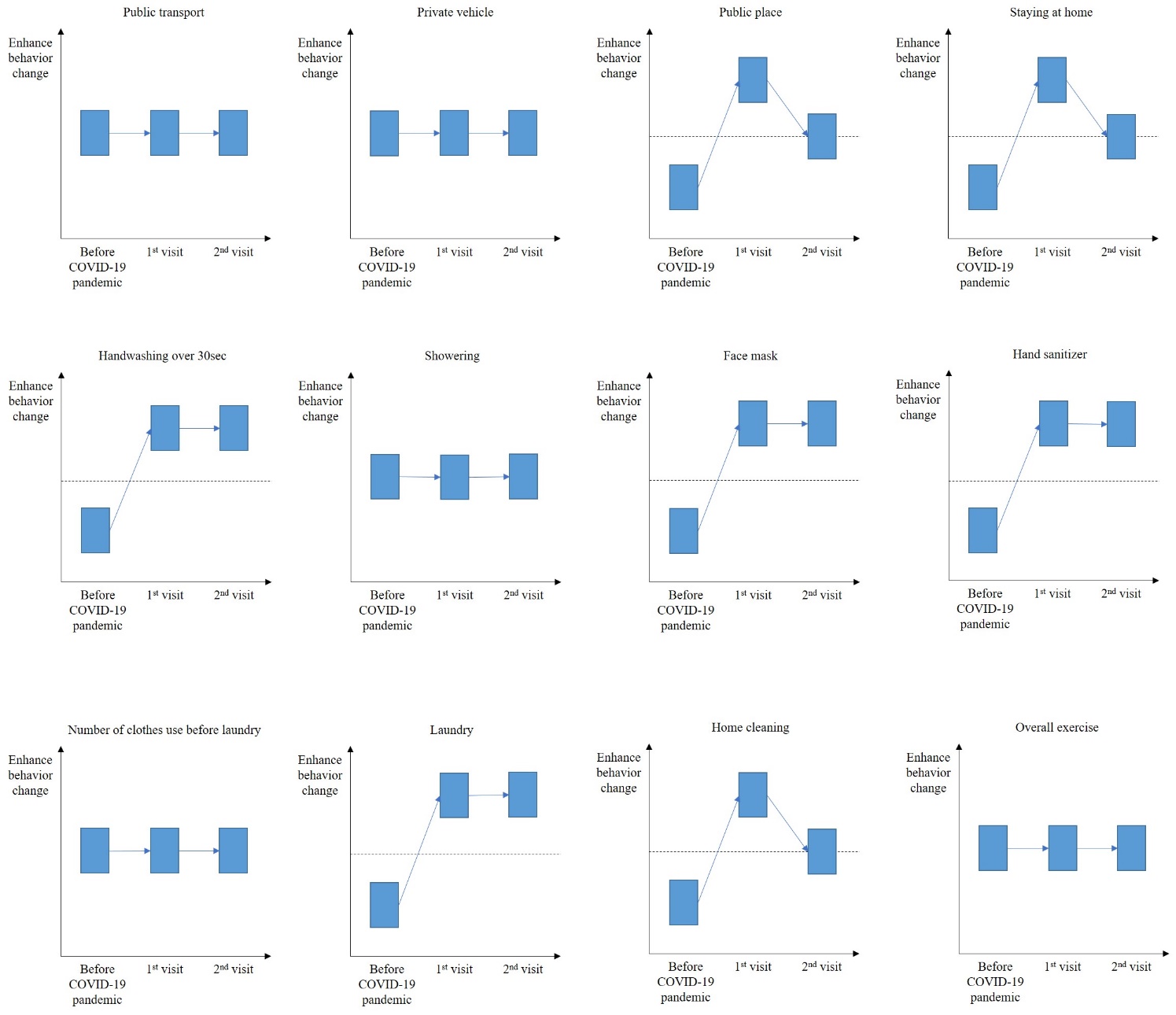


Figure S7. Number of second visit participants of study and COVID-19 cases in South Korean by day


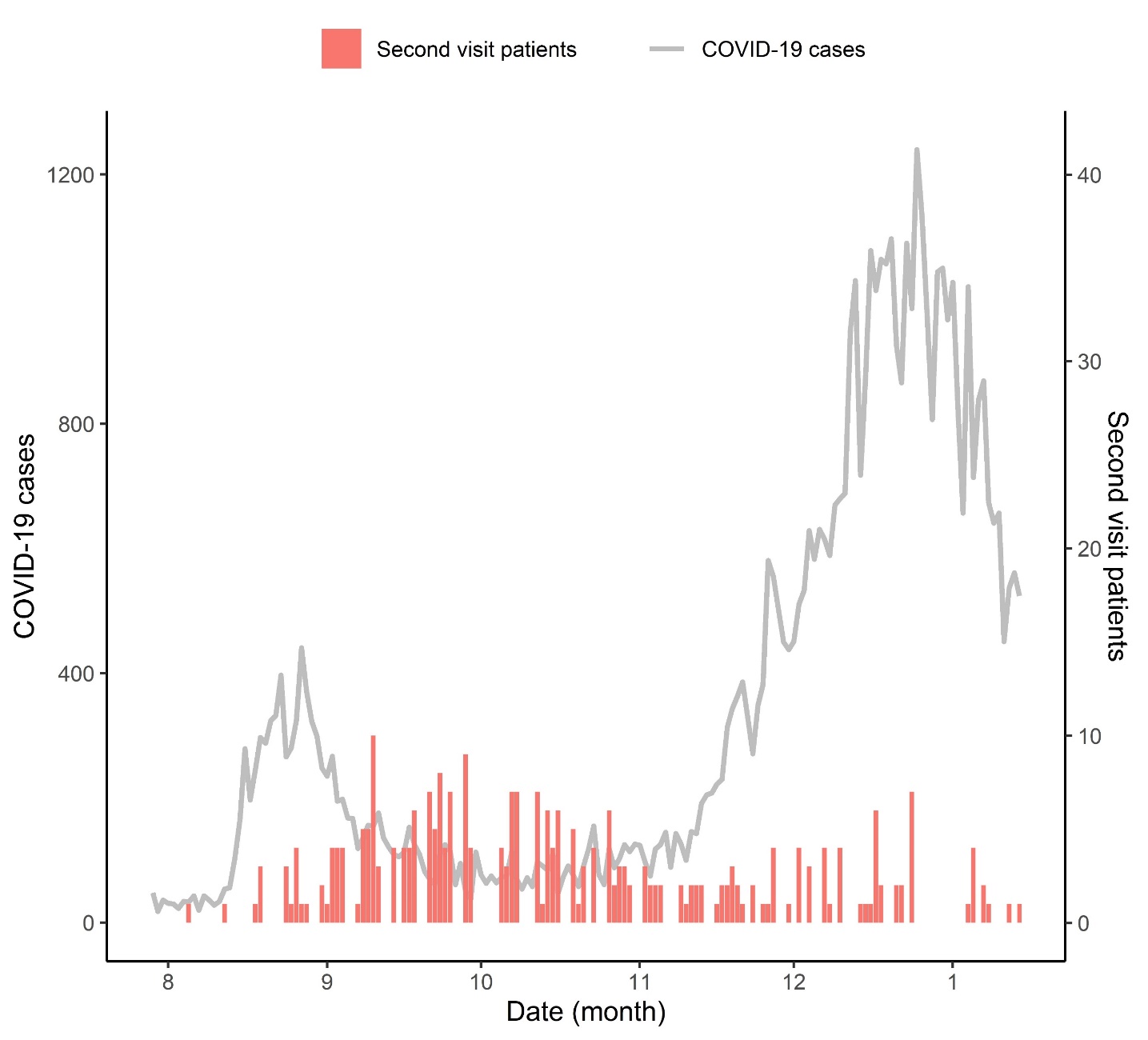

Supplement: Supplementary file 1 — Supplementary Information. [file 41598_2022_19787_MOESM1_ESM.docx]
